# The Use of Simulators in Training for Bovine Reproductive Procedures: A Scoping Review

**DOI:** 10.3390/ani16010140

**Published:** 2026-01-04

**Authors:** Heitor Azuaga Filho, Bruno Colaço, Rita Payan-Carreira

**Affiliations:** 1 Instituto Federal de Educação, Ciência e Tecnologia de Mato Grosso-Campus Cáceres, Cáceres 78201-382, Brazil; heitor.filho@ifmt.edu.br; 2 Veterinary and Animal Research Centre (CECAV), University of Trás-os-Montes and Alto Douro (UTAD), 5000-801 Vila Real, Portugal; 3 Associate Laboratory for Animal and Veterinary Sciences (AL4AnimalS), University of Trás-os-Montes and Alto Douro (UTAD), 5000-801 Vila Real, Portugal; 4 Comprehensive Health Research Centre (CHRC), Universidade de Évora, 7004-516 Évora, Portugal; 5 Departmento de Medicina Veterinária, Escola de Ciências e Tecnologia, Universidade de Évora, 7004-516 Évora, Portugal

**Keywords:** bovine reproductive procedures, simulation-based training, bovine reproductive simulators, artificial insemination, transrectal palpation, competency-based education

## Abstract

Training in bovine reproduction requires not only technical proficiency but also ethical responsibility and adherence to animal-welfare standards. Traditional instruction relies heavily on repeated practice in abattoir-collected specimens and live cattle, raising concerns about stress, variability, logistical constraints, and student anxiety. Simulation-based education (SBE) has therefore emerged as a pedagogically robust and ethically sound complement to clinical teaching, enabling learners to acquire psychomotor and cognitive skills in structured, low-risk environments. This scoping review synthesizes current evidence on validated simulators used to train bovine reproductive procedures, with particular emphasis on artificial insemination, transrectal palpation, and pregnancy diagnosis. Following Arksey and O’Malley’s framework, a comprehensive search of three international databases identified 13 eligible studies that described simulator typologies, validation approaches, implementation strategies, and educational outcomes. Simulators ranged from low-cost handmade models to high-fidelity haptic and hybrid systems, each offering distinct advantages across diverse instructional contexts. Evidence consistently showed that simulator-based training improves anatomical orientation, technical performance, procedural efficiency, and learner confidence, while reducing anxiety and the need for novice practice on live cattle. However, validation standards remain inconsistent, long-term transfer to clinical practice is poorly documented, and most commercial models inadequately represent *Bos indicus* anatomy, limiting global applicability. Simulation can substantially strengthen competency-based animal and veterinary curricula and advance the 3Rs by replacing or refining early live-animal procedures. To fully realize this potential, future efforts should prioritize rigorous validation, greater anatomical representativeness, and improved accessibility through modular, low-cost designs. Simulation-based training thus represents both an educational innovation and an ethical imperative in modern veterinary practice.

## Simple Summary

Training in bovine reproductive procedures is essential for animal health, food production, and welfare. Traditionally, students learned these skills directly on live animals, but this approach raises ethical concerns, depends on animal availability, and poses safety risks for both learners and animals. Addressing these challenges, training simulators have been developed, allowing students to practice procedures in controlled, humane environments before working with live cattle. This scoping review systematically examined the scientific literature to identify available simulators for teaching cattle reproductive skills, assess their anatomical accuracy, evaluate validation methods, and explore their contribution to ethical training practices. The findings reveal that, while numerous simulators exist and are widely adopted in veterinary education, most lack rigorous validation based on objective learning outcomes and instead rely primarily on user satisfaction surveys. Additionally, breed-specific anatomical variations and real-world clinical conditions are often inadequately represented in current simulator designs. Despite these limitations, simulators demonstrate strong potential to enhance skill acquisition while reducing reliance on live animals, aligning with ethical principles of animal use in education. However, further research is needed to establish their educational effectiveness and ethical impact. This evidence-based synthesis guides educators, institutions, and policymakers seeking to improve veterinary training while prioritizing animal welfare.

## 1. Introduction

### 1.1. Simulators and Competency Based Training in Veterinary Education

Animal and Veterinary education has progressively shifted toward competency-based frameworks in which graduates must demonstrate clearly defined learning outcomes related to technical proficiency, clinical reasoning, animal welfare, and ethical practice [1,2]. In bovine theriogenology, core competencies include safe and effective execution of reproductive procedures such as artificial insemination (AI), transrectal palpation, ovarian and uterine evaluation, and pregnancy diagnosis [3]. These competencies are technically demanding and require structured, progressive training pathways.

Traditionally, the acquisition of bovine reproductive skills relied extensively on repeated practice in live cattle though simulators are also gaining popularity as supplementary training tools [3,4]. Although such experiences offer authentic clinical exposure, they are associated with well-documented challenges: limited and sometimes unpredictable access to suitable animals; variability in anatomical or physiological conditions; safety risks for inexperienced learners [3]; along high levels of stress or anxiety among students encountering invasive procedures for the first time [5,6,7,8,9]. These constraints also hinder standardization of teaching activities and complicate the implementation of objective assessment strategies.

Human medical education faced similar challenges decades earlier, prompting substantial innovation in simulation-based education (SBE). These developments also translated to the curricular contexts of animal and veterinary sciences [5,10,11]. A robust body of evidence now demonstrates that simulation enhances psychomotor and non-technical skills, supports deliberate practice, and improves patient safety [12]. These principles have increasingly been translated to the sector of animal and veterinary training, where simulators are being adopted to scaffold learning progression from low-stakes environments to supervised clinical encounters [9,10]. Moreover, approaches such as peer-assisted learning utilizing simulators have been explored to optimize curriculum delivery [13].

Training in bovine reproductive procedures occurs across multiple educational stages, including undergraduate veterinary and zootechnical curricula, postgraduate specialization programs, and early professional practice. Animal availability, welfare concerns, and institutional resources often limit opportunities for repeated exposure to live transrectal palpation during undergraduate training. These constraints reinforce the pedagogical value of simulators as tools to ensure baseline procedural competence before clinical exposure.

Within bovine reproduction, simulators such as Breed’n Betsy (Brad Pick-ford, Byaduk, Victoria, Australia) and the Haptic Cow (University of Glasgow, UK) exemplify how SBE can strengthen the learning process. These devices allow learners to explore reproductive anatomy, practice palpation techniques, and rehearse AI maneuvers while receiving guided or automated feedback [14,15,16]. Their integration enables instructors to design intentional practice sequences aligned with explicit competencies, helping students develop spatial understanding and procedural fluency before transitioning to live-animal practice.

### 1.2. Educational Rationale for Simulation Based Training

The pedagogical rationale for using simulators aligns with established learning theories of adult education, which stress the importance of practical, experience-driven learning opportunities [17], as well as psychomotor development, by providing repetitive practice opportunities, a key component of motor learning [18], particularly relevant in procedures requiring fine motor control and precision [19,20]. Simulation provides a structured environment for learners to engage in deliberate practice, a learning model characterized by repetition, targeted feedback, and progressive difficulty [8,14,21,22]. This learning model is essential for mastering tactile procedures such as transrectal palpation or pregnancy diagnosis in bovine, which rely heavily on haptic perception, spatial mapping, and memory consolidation.

Simulators also provide a safe space for failure [11,23], where learners can make mistakes and learn from them without compromising animal welfare or creating negative emotional experiences [7,9,24], for themselves or the animals [25]. This psychological safety is critical in early theriogenology teaching, as students often report apprehension, discomfort, or fear of causing harm during their initial interactions with animals [14,26,27].

Simulators can further contribute to the assessment of procedural skills and support constructive alignment by enabling educators to define explicit learning outcomes, such as “perform AI in a cow safely and effectively” or “deposit semen in the uterine body of a cow within a specified timeframe,” and to design simulator-based activities that address component skills. Performance can then be assessed using structured rubrics or objective structured clinical examinations (OSCEs), ensuring coherence between teaching methods and intended competencies.

From an ethical perspective, integrating SBE contributes to a more humane and responsible approach to veterinary training. By reducing the number of initial novice attempts performed on live cattle, simulators directly support the principles of the 3Rs—Replacement, Reduction, and Refinement—which guide the ethical use of animals in education [28]. Their use also provides a platform for explicit discussions about welfare, empathy, and professional responsibility [29,30], encouraging students to reflect on the sensory and emotional experiences of animals subjected to reproductive procedures.

Taken together, the educational and ethical arguments strongly support the integration of simulators into competency-based veterinary curricula. Their strategic use allows learners to build foundational conceptual, spatial, and psychomotor skills before engaging in more complex, real-world clinical contexts, thereby enhancing both technical proficiency and animal welfare standards.

Despite growing recognition of the educational value of simulators in bovine theriogenology, their implementation remains heterogeneous across institutions, and critical questions regarding their design, validation, and pedagogical integration remain inadequately addressed. While previous reviews have examined simulation-based education in veterinary training more broadly [5,8], no comprehensive synthesis has systematically evaluated how bovine reproductive simulators are validated for educational effectiveness, how accurately they represent breed-specific anatomical variations, or how their use aligns with contemporary ethical frameworks such as the 3Rs principles.

To address these gaps, this scoping review systematically maps the current landscape of bovine reproductive simulators with explicit focus on three interrelated dimensions: (1) validation strategies employed to establish educational and clinical fidelity; (2) anatomical representativeness, particularly regarding breed-specific reproductive tract morphology; and (3) ethical implications within competency-based veterinary curricula. By synthesizing evidence across these domains, this review provides the first comprehensive, evidence-based framework to guide informed curricular design, institutional investment decisions, and future research priorities in simulation-based bovine theriogenology education.

## 2. Methodology

This scoping review was designed to map and synthesize current evidence on the use of simulators for training bovine reproductive procedures, with particular attention to educational outcomes, implementation models, and validation strategies. The methodological framework followed the five-stage approach proposed by Arksey and O’Malley [31]: identification of research questions, identification of relevant studies, study selection, data charting, and synthesis. This structure ensured a transparent and replicable process.

### 2.1. Rationale and Research Questions

The review was motivated by an apparent gap in the literature: although simulators have been increasingly incorporated to train and develop procedural skills in bovine reproduction, rigorous validation studies examining their educational effectiveness remain limited. Consequently, this exploratory scoping review aimed to systematically map the current use of simulation-based training practices in bovine reproduction and to contextualize findings within broader discussions on animal welfare and pedagogical innovation in zootechnical and veterinary education. Specifically, we examined the educational contexts in which simulators are used, the learning outcomes they support, and the forms of validation applied to these tools. No protocol was registered a priori (e.g., OSF or PROSPERO), consistent with standard scoping review practice for exploratory evidence mapping and because this study was designed to capture a broad and evolving evidence base rather than to test predefined hypotheses or conduct a quantitative synthesis.

Three research questions guided this review:

1. *Which simulators have been described to support training in reproductive technical procedures?*
2. *What evidence exists regarding the effectiveness of simulator-based learning for these procedures?*
3. *What types of formal validation have been reported for these simulators?*

### 2.2. Inclusion and Exclusion Criteria

Study selection was guided by the SPIDER framework [32]: Sample/Population (type of participants); Phenomenon of Interest (reports on simulator use for training bovine reproductive procedures); Design (description of the intervention); Evaluation (assessment of the learning outcomes and simulator validation); Research Type (original reports of interventions). This framework was selected for its suitability in reviews addressing qualitative and mixed-methods evidence commonly found in educational research.

Eligible studies met the following criteria: (1) original research articles and PhD theses; (2) description of simulator development, implementation, validation, or evaluation in educational settings; (3) focus on teaching bovine reproductive skills using simulators; (4) comparison of different simulators for training technical procedures; (5) preference for peer-reviewed publications; (6) full-text availability; (7) publications in English, Spanish, or Portuguese; studies in other languages were translated for screening when necessary.

Studies were excluded if they (1) were review articles; (2) focused primarily on basic reproduction research without an educational component; (3) addressed topics outside the reproduction domain or unrelated species; (4) were conference abstracts, posters, or book chapters without complete original data; or (5) were limited to live-animal or cadaver-based teaching without simulators or synthetic models. Detailed criteria are presented in Table 1.

### 2.3. Search Strategy

A comprehensive search was conducted across major databases relevant to veterinary and animal sciences: PubMed, Scopus, and Web of Knowledge (Clarivates). The search strategy was iteratively refined to balance sensitivity and specificity. Keywords and expressions related to simulators, veterinary or animal education, and bovine reproductive procedures were combined to retrieve relevant publications. Search phrases included “veterinary simulators reproduction teaching”, “bovine reproduction simulator education”, and “theriogenology simulators”. Boolean operators and truncated terms were explored but ultimately discarded, as these refinements did not improve search yield.

To ensure comprehensive retrieval, a supplementary hand search was conducted in Google Scholar using identical search terms to capture potentially relevant studies from non-indexed sources. No date limits or language restrictions were applied during the initial search. Articles published in languages other than English, Spanish, or Portuguese were translated using Google Translate prior to screening. The use of Google Scholar as a supplementary source entails important methodological constraints (e.g., lack of transparency in indexing criteria, algorithm-driven ranking that may vary over time, and reduced reproducibility of search results) that might introduce selection bias and limit systematic control over the retrieval process. To mitigate these limitations, Google Scholar results were treated exclusively as a complementary source, and all retrieved records were screened using the same predefined eligibility criteria applied to indexed databases.

The final search was completed on 23 June 2025, establishing the upper limit for study inclusion.

### 2.4. Study Selection and Data Extraction

**Figure 1:**
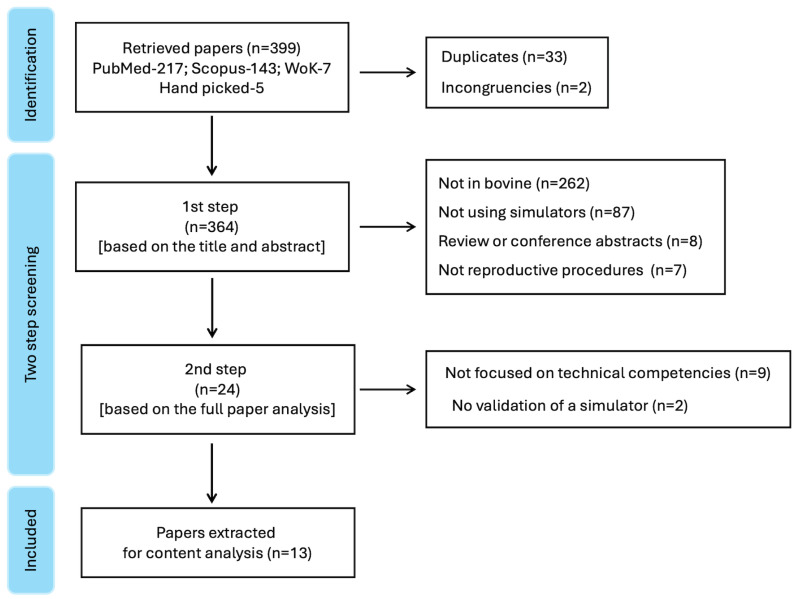
Flow diagram detailing the screening process. WoK-Web of Knowledge (Clarivate).

The initial search retrieved 399 papers, which underwent a two-stage filtering process. First, 35 duplicates and non-functional records (e.g., URLs not linking to articles) were removed. Titles and abstracts of the remaining studies (*n* = 364) were independently screened by two reviewers using the predefined criteria. Articles deemed potentially relevant (*n* = 24) proceeded to full-text assessment. Discrepancies in eligibility decisions were resolved through consensus discussion. Ultimately, thirteen articles met all inclusion criteria and were included in the review (Figure 1).

A structured data extraction form was developed to ensure systematic and comprehensive charting of relevant study characteristics. Extracted variables included: simulator type and description; fidelity level, components, and cost (when reported); study design; participant characteristics (e.g., training level, program context, number of participants, prior experience); comparison or control conditions; intervention features (training duration, number of sessions, curricular integration); outcome measures (objective performance indicators and subjective learner feedback); validation strategies employed; principal quantitative findings; follow-up data; funding sources or potential industry involvement; and authors’ key conclusions. This multifaceted approach enabled comprehensive examination of both technical and educational dimensions of simulator use.

### 2.5. Data Analysis and Synthesis

Given the heterogeneity of study designs, simulator types, and outcome measures, findings were synthesized narratively rather than through quantitative meta-analysis. Studies addressing the same simulator or instructional approach were compared to identify commonalities, methodological patterns, and recurring gaps.

The analysis focused on five thematic domains: (1) simulator typology and fidelity; (2) educational implementation strategies; (3) learning outcomes and skill transfer; (4) validation approaches and quality of evidence; (5) ethical and welfare considerations. Particular attention was paid to alignment between training activities and assessment methods and to whether studies addressed long-term retention or transfer to clinical performance—key indicators of durable learning in procedural training.

## 3. Results

The thirteen included studies (Supplementary Table S1) were published between 2005 and 2025, encompassing diverse educational contexts and simulator types. Participant populations ranged from early-stage veterinary students with no prior procedural experience to final-year students, AI technicians, and experienced veterinarians. Training interventions varied considerably, from single short sessions to intensive multi-week programs, with group sizes ranging from individual practice to cohorts of up to 72 students.

### 3.1. Simulator Typology and Technical Characteristics

Simulators for bovine reproduction can be grouped into three main categories: physical models, virtual and haptic systems, and hybrid designs. These categories differ in fidelity, technology, and resource requirements. As summarized in Table 2, simulators used for training in bovine reproduction varied in sophistication from low-cost handmade physical models to advanced virtual reality systems with haptic feedback. Each typology offers distinct advantages depending on institutional resources, learning objectives, and anatomical specificity.

Six distinct simulator types were identified in the reviewed literature, classified by technology platform and fidelity level (Table 2). Physical models represented the majority: Breed’n Betsy, Simulador Bovino F1, and SIMCA-COW. Haptic/virtual reality systems included the Haptic Cow and stand-alone PHANToM device. A hybrid category encompassed the TrAI4Nel system, which combined physical and electronic components.

Physical models remain the most common. Full-sized physical simulators such as Breed’n Betsy consisted of a steel or aluminum frame with an artificial vulva, anal sphincter, and replaceable latex reproductive tract components. This model permitted palpation at multiple gestational stages by exchanging latex uterine replicas [21,26,33,34].

These models allow students to familiarize themselves with reproductive structures, practice transrectal palpation, and rehearse pregnancy diagnosis within a controlled environment. The Simulador Bovino F1 featured a life-sized fiberglass shell with latex reproductive tract components representing external bovine landmarks and internal structures [35]. The SIMCA-COW represented a low-cost alternative, constructed from bovine pelvis with rigid support frames and interchangeable slaughterhouse-derived uteri, costing less than USD 40 [27]. These three models offered variable tactile resolution and durability, with SIMCA-COW requiring manual assembly and periodic replacement of biological components.

Virtual and haptic systems include the Haptic Cow that combines three-dimensional virtual anatomies with force-feedback interfaces that simulated tissue resistance and spatial constraints [4,14]. This high-fidelity platform enabled guided navigation of internal reproductive structures and exploration of anatomical scenarios difficult to replicate in physical models. The stand-alone PHANToM device employed a haptic arm interface with automated capability for independent user practice [15].

The TrAI4Nel simulator—an hybrid system—integrates physical components (silicone cervix with double-layer construction) with electronic sensors and Arduino-controlled peristaltic motion, providing real-time visual feedback via LED indicators. This system is anatomically specific for *Bos indicus* [22,36].

### 3.2. Procedures Trained and Educational Applications

The thirteen studies documented training for four primary procedural competencies: transrectal palpation, pregnancy diagnosis, artificial insemination catheter placement, and reproductive ultrasonography. Most simulators (*n* = 11) addressed transrectal palpation, with emphasis on anatomical structure identification, particularly the cervix, uterus, and ovaries [4,14,15,26,35]. Three studies incorporated pregnancy diagnosis training [21,33,34], one included ultrasonographic evaluation [27], and one focused exclusively on artificial insemination catheter placement [22].

Despite heterogeneity in simulator platforms, educational implementation followed a relatively consistent pedagogical logic across most studies. A typical progression consisted of: (1) theoretical instruction in reproductive anatomy and physiology, (2) hands-on simulator practice for tactile exploration and procedural rehearsal, and (3) supervised clinical practice with live animals [14,15,22,26,27,35]. In one exception [34], simulators were employed as a substitute rather than precursor to live animal practice. Within this scaffolded framework, simulators served complementary functions: familiarization with pelvic cavity anatomy, repeated procedural rehearsal, anxiety reduction before live animal encounters, and standardized learning experiences independent of animal availability.

Beyond skills training, simulators were applied to competency-based assessment. Annandale et al. [33] demonstrated that simulator-based scores in pregnancy diagnosis predicted subsequent accuracy in live cows, supporting the potential of these tools for reproducible, objective evaluation amenable to objective structured clinical examination (OSCE)-style formats.

### 3.3. Educational Implementation Strategies

Simulators for bovine reproductive training have been incorporated into diverse educational settings, ranging from undergraduate courses in veterinary medicine to professional continuing education and technician certification programs (Table 3).

***Study Designs and Participants***

The extracted studies employed diverse methodological designs reflecting different educational research questions and institutional contexts. Designs included controlled experiments comparing training sequences, pre-post designs measuring within-group changes, observational studies, and cohort studies. Participants represented broad learner populations (Table 3): veterinary students ranging from preclinical beginners with no palpation experience [4,14,34] to final-year students preparing for practice [16,21]; artificial insemination technician trainees [22]; and experienced veterinarians serving as expert evaluators or validation study subjects [22].

***Training Implementation Models***

Implementation approaches spanned a spectrum from highly structured instructor-led sessions to autonomous self-directed practice. Instructor-led models included Breed’n Betsy [16,21,34], Simulador Bovino F1 [35], SIMCA-COW [27], and TrAI4Nel [22], where educators monitored student performance and provided real-time feedback. In contrast, automated versions of the Haptic Cow eliminated the need for continuous instructor presence, enabling independent practice guided by expert-recorded computer protocols [15,26].

Group sizes and training duration varied substantially (Table 4). Groups ranged from individual practice sessions [21] to cohorts of 10 students [22], while duration extended from single sessions [21] to 4 h simulator sessions preceded by 2 h of anatomical instruction [35] or 8h of simulator training alternating in a cross pattern with training with abattoir specimens [22]. Training intensity also differed markedly: one study compared an intensive simulator protocol (25 palpations over brief training) to distributed live animal practice (>200 examinations) over an academic year [34].

***Curricular Integration and Pedagogical Approaches***

In most interventions, simulation sessions were integrated as structured practical modules within reproductive physiology or theriogenology courses. Learners first received theoretical overviews of the topic of concern, followed by hands-on simulator practice. Training typically employed progressive task difficulty, permitting repeated procedural rehearsal under controlled conditions [22,26,27]. This scaffolded approach allowed students to acquire tactile familiarity and procedural confidence before transitioning to live animals, thereby promoting skill consolidation while reducing potential animal welfare concerns.

Interactive feedback mechanisms enhance learning in advanced systems. The TrAI4Nel simulator introduced electronic sensors that signaled incorrect cervical passage or semen deposition, monitoring autonomous performance and enabling immediate error correction without instructor intervention [36]. The automated Haptic Cow allowed independent practice with computer-guided instruction [4,15]. These feedback systems represent an emerging pedagogical trend toward autonomous or blended learning models in veterinary technical skill acquisition.

Beyond training, some studies structured simulator use as standardized assessment through sequential skill stations within OSCE formats, with performance scored using standardized rubrics [33]. Others employed simulators to measure objective improvements such as reductions in procedure time and increases in learner confidence over successive attempts [27]. Implementation strategies are summarized in Table 4, which details procedures trained, study populations, instructor involvement, and assessment approaches across all thirteen studies.

### 3.4. Learning Outcomes and Training Effectiveness

***Objective Performance Measures***

Simulator training consistently produced improvements in objective performance indicators across multiple competency domains. For structural identification, the Haptic Cow demonstrated superior uterus location and identification accuracy compared to traditional instruction alone [14], while the Simulador Bovino F1 produced better anatomical structure recognition than conventional training [35]. However, performance outcomes were heterogeneous. In the study by da Silva & Pinto [27], all students successfully palpated basic uterine structures following simulator training, but only 62.5% successfully located the cervix or identified ovarian follicles, and 37.5% identified the corpus luteum via ultrasonography.

For artificial insemination procedures, the SIMCA-COW enabled a 55% reduction in catheter insertion time from first to fourth attempt [27], and TrAI4Nel training increased AI success rates in live cows and reduced the number of attempts required for successful procedure completion [22].

***Subjective and Confidence-Based Outcomes***

Student confidence and perceived preparedness improved consistently across simulator training interventions. Multiple studies documented increased confidence [22,26,27], particularly when training occurred in small groups of 3–5 students. Simulator training also produced more realistic self-assessment and improved understanding of reproductive anatomical locations compared to theoretical instruction alone [26,27]. Learners consistently perceived simulators as safe practice environments permitting repeated rehearsal and risk-free failure. Educators valued simulators’ capacity for standardized assessment, objective performance tracking, efficient institutional animal resource utilization, and facilitation of peer-assisted and team-based learning approaches.

***Pregnancy Diagnosis***

Pregnancy diagnosis presents a more complex picture than basic palpation or artificial insemination, with apparent contradictions between studies favoring simulator training and those demonstrating advantages of live animal instruction. The Breed’n Betsy simulator exhibited limitations for early pregnancy detection (<6 months gestation), with simulator-trained students achieving lower sensitivity than live-trained cohorts; however, sensitivity was equivalent for late pregnancy (>6 months) [21]. This differential performance likely reflects mechanical constraints rather than pedagogical inadequacy: latex organs and wooden disk representations of cotyledons cannot fully replicate the subtle texture variations characteristic of early pregnancy, whereas pronounced uterine dimensional changes in late pregnancy are more readily simulated. Bossaert et al. [34] demonstrated that live cow training significantly outperformed Breed’n Betsy training for ovary localization and evaluation. Notably, this study found that 25 simulator palpations alone were insufficient for consistent expertise, indicating that simulators provide foundational skill development but cannot entirely substitute for extensive live animal practice.

### 3.5. Validation of Simulators

Although multiple forms of validation are reported, most simulators have been assessed primarily through face validity and user perception measures. Evidence supporting construct and criterion validity remains limited, and in several cases is derived from small, context-specific cohorts. Consequently, claims regarding educational effectiveness, ethical benefit, or clinical transfer should be interpreted cautiously, as they are not uniformly supported by robust empirical data. As simulator diversity and educational applications have expanded, validation efforts have increased correspondingly. However, most commercial simulator models lack formal scientific validation or remain insufficiently validated. Formal validation determines whether training with a simulator translates into improved technical performance on live animals and whether procedural skills acquired during simulation transfer to real clinical practice. This assessment is particularly critical for complex procedures requiring psychomotor skills and physical movements performed blindly within the animal, intertwined with cognitive processes. Validation is central to justifying simulators’ inclusion as core curricular resources.

The reviewed literature reported simulators validated through multiple approaches, with most studies employing performance-based comparative designs measuring skill transfer and learning outcomes. However, only a small proportion of theriogenology-related simulators have undergone formal validation in the published literature, as commented by Koziol et al. [11]. Table 5 synthesizes the formal validation evidence available for each simulator platform, demonstrating significant heterogeneity in validation completeness and rigor.

***Face, Content, and Construct Validity***

Most simulators were validated at elementary validity levels, typically assessed via self-reported questionnaires. Face validity (whether a simulator appears realistic and relevant to the procedural task) was demonstrated for Breed’n Betsy [21,34], SIMCA-Cow [27], Haptic Cow/PHANToM [4,14,15], and TrAI4Nel [22]. Many commercial models have been replicated and adopted primarily based on user satisfaction and appeal without formal validation, or as a protective measure for animal welfare in teaching contexts.

Content validity (comprehensive incorporation of necessary anatomical structures, procedural steps, and clinical scenarios) was examined for the Haptic Cow [14,15], SIMCA-Cow [27], and TrAI4Nel [22]. Construct validity (ability to discriminate between different skill levels and/or measure claimed clinical competencies) was reported only for Breed’n Betsy [21] and TrAI4Nel [22].

***Criterion Validity***

Criterion validity—encompassing concurrent validity (correlation between simulator performance and contemporaneous measures of clinical competence) and predictive validity (ability to forecast future real-world technical performance)—holds particular interest for educators. Criterion validity was established for the Haptic Cow [14,15], demonstrating that simulator training improved uterus identification accuracy in live animal examinations. Annandale et al. [33] demonstrated that OSCE scores obtained using Breed’n Betsy predicted pregnancy diagnosis performance several months later. TrAI4Nel showed predictive validity with improved live animal AI success rates following simulator training [22]. No formal validation data were reported for Simulador Bovino F1.

## 4. Current Limitations and Challenges

Despite technological advances in simulator design, bovine reproductive simulators face multiple interconnected limitations that impede their optimal integration into veterinary curricula. These constraints encompass technical fidelity barriers, validation gaps, economic factors, and anatomical specificity issues. Addressing these challenges is essential to realize the full potential of simulation-based training in bovine reproduction.

### 4.1. Fidelity and Technical Limitations

Current simulator designs struggle to replicate the complex tactile feedback and tissue consistency inherent to live animal examinations. While functional fidelity—the degree to which simulator responses match clinical reality—is recognized as paramount for skill transfer [37], existing platforms exhibit measurable gaps in replicating tissue properties and anatomical variability.

The Breed’n Betsy simulator exemplifies these constraints. Bossaert et al. [34] observed that students trained exclusively on this device experienced greater orientation difficulties during subsequent live animal procedures, suggesting that simulator-based experience alone may not fully prepare learners for clinical variability. For pregnancy diagnosis specifically, Breed’n Betsy demonstrated lower sensitivity for early bovine pregnancies (<6 months gestation) compared to live animal training [21], despite being purpose-designed for this procedure. This limitation reflects mechanical rather than pedagogical constraints: latex organs and wooden disk representations of cotyledons cannot replicate the subtle texture variations characteristic of early pregnancy. Late pregnancies, characterized by pronounced uterine dimensional changes, transferred more readily from simulator to clinical settings.

Ongoing development of biomaterials and haptic systems remains essential to enhance tactile realism. However, balancing improved fidelity with practical constraints—including durability, cost, and maintainability—presents a persistent design challenge. The complexities of maintaining educational effectiveness while addressing animal welfare and ethical considerations further constrain simulator development trajectories [38,39].

### 4.2. Validation and Standardization Deficits

A critical limitation of the current simulator landscape is the paucity of rigorous validation evidence. Most commercial bovine reproductive simulators have undergone only subjective face validity assessments, inadequately characterizing their educational effectiveness relative to substantial acquisition costs. Koziol et al. [11] surveyed simulator models identified by veterinary educators and found that only one had undergone formal validation reported in peer-reviewed literature. This gap represents a significant barrier to evidence-based tool selection and curricular integration.

The absence of industry standards and manufacturing quality variability further impede cross-platform comparison and benchmarking. Without standardized validation protocols, educators lack objective criteria for discriminating between simulator platforms or justifying investment decisions. Additionally, the assumption that simulator similarity necessarily confers equivalent learning outcomes—evident in the market proliferation of models resembling Breed’n Betsy or other established designs—remains empirically unsupported and may mask important functional or pedagogical differences between platforms.

Establishing consensus on validation standards and reporting requirements would address this limitation and facilitate accumulation of evidence regarding simulator effectiveness across different educational contexts and learner populations.

### 4.3. Economic Barriers to Adoption

Cost, a substantial practical limitation to widespread simulator adoption, represent a significant differentiating factor across simulator categories. High-fidelity systems such as the Haptic Cow [14] and hybrid platforms require significant capital investment and ongoing maintenance, effectively restricting access to well-resourced institutions and limiting individual student practice opportunities. Increased maintenance costs were anticipated for simulators used in artificial insemination training compared to transrectal palpation models, due to material fatigue from catheter abrasion [27]. The economic barrier is compounded by the consumable nature of simulator components: repetitive use necessitates regular replacement of worn surfaces and anatomical elements, progressively affecting model accuracy and cumulatively increasing operational costs.

Conversely, low-cost alternatives such as SIMCA-COW required minimal investment (<USD 40), although they involved manual assembly and ongoing material replacement, demonstrating that effective training tools can be developed for resource-constrained contexts [27]. However, such models often compromise specific fidelity aspects, including tactile resolution and anatomical precision, and are also more susceptible to biosecurity issues. The trade-off between cost and fidelity thus remains unresolved, with resource availability ultimately determining simulator type accessibility across diverse institutional settings globally.

### 4.4. Anatomical Specificity and Breed Representation

Most commercially available bovine reproductive simulators predominantly represent *Bos taurus* cervical anatomy. This narrow representation poses challenges for professionals and trainees working with *Bos indicus* breeds such as Nelore cattle, which exhibit distinct cervical morphology including longer and more tortuous cervices requiring substantially different manipulation techniques. The limited anatomical diversity in available simulators reflects the geographical concentration of simulator research and development, with models optimized for specific regional breeding systems rather than global livestock diversity.

Currently, only TrAI4Nel has been specifically designed for *Bos indicus* cervical anatomy [36], representing an isolated instance of breed-specific simulator development. Expanding anatomical representation to encompass diverse bovine breeds would enhance global applicability and contextual relevance of training tools, particularly benefiting practitioners in tropical and subtropical regions where Bos indicus genetics predominate. This expansion requires collaborative development efforts involving researchers and educators from underrepresented geographical regions.

## 5. Future Directions

Simulation-based training in bovine reproduction stands at a critical juncture, with emerging technologies and pedagogical innovations offering pathways to enhanced learning outcomes, broader accessibility, and stronger alignment with animal welfare imperatives. Realizing these opportunities requires coordinated progress across five interconnected domains: technological advancement, pedagogical innovation, economic accessibility, anatomical diversity, and validation rigor.

### 5.1. Artificial Intelligence and Adaptive Learning Systems

Artificial intelligence-driven feedback represents a promising avenue for personalizing skill acquisition. AI systems capable of analyzing learner performance metrics—including applied force, procedural trajectory, step duration, and error patterns—could function as adaptive tutors, automatically adjusting scenario difficulty and providing real-time individualized guidance [40]. Such systems would enable self-paced learning pathways tailored to individual competency gaps, potentially accelerating progression from novice to expert performance levels.

Beyond training, AI-generated analytics could establish objective competency thresholds, distinguishing proficiency levels through comprehensive assessment of time efficiency, precision, force application, and success rates. This standardized approach to performance benchmarking would support evidence-based certification and quality assurance in veterinary training programs. However, implementation requires careful validation to ensure that AI-derived performance criteria align with clinically relevant competencies and do not introduce algorithmic biases that disadvantage learners from different backgrounds or training contexts.

### 5.2. Enhanced Haptic and Sensory Fidelity

Next-generation haptic systems and advanced biomaterials hold substantial promise for replicating the subtle tactile nuances critical to clinical reproductive examination. Future simulator development should prioritize incorporation of variable resistance patterns reflecting different estrous cycle phases, vascular pulsations mimicking physiological structures, dynamically responsive textures indicating pathological conditions, and systemic parameters such as body temperature and peristaltic movements.

Intelligent biomaterials capable of adapting to user interactions while maintaining durability under repetitive use represent a necessary prerequisite for achieving high-fidelity experiences that reliably transfer to live-animal practice. Parallel advances in materials science and haptic engineering will be essential to balancing tactile realism with practical constraints of cost, durability, and component replaceability.

### 5.3. Immersive and Collaborative Learning Platforms

Virtual Reality (VR) and Augmented Reality (AR) technologies offer expanded opportunities for immersive learning environments [41]. These platforms can facilitate detailed three-dimensional anatomical visualization, complex decision-making training in simulated field conditions—including management of animal behavior and adverse environmental factors—and geographically distributed collaborative training among students and practitioners. Integration of VR/AR technologies with physical simulators could create blended learning environments that combine tactile skill development with cognitive and situational awareness training, addressing the multifaceted competencies required for contemporary veterinary practice.

Early adoption of these technologies in veterinary education demonstrates feasibility, though questions remain regarding cost-effectiveness, learning curve requirements for novice users, and optimal integration with traditional curricula.

### 5.4. Modular Design and Anatomical Diversity

Development of modular simulator designs with interchangeable anatomical components represents a strategic approach to enhancing versatility while managing costs. Systems such as TrAI4Nel [36] or Henryetta [42], featuring replaceable cervical components, exemplify how anatomical diversity can be incorporated through component substitution rather than complete simulator replacement. This modularity enables adaptation across different bovine breeds—particularly addressing the critical gap in *Bos indicus* representation—and accommodation of progressive complexity levels suited to learners at various training stages.

Advances in biomaterials engineering should prioritize development of durable, cost-effective components that maintain tactile fidelity through extended use cycles. Expanding anatomical representation to encompass cattle genetic diversity remains a pressing priority for ensuring global relevance and equitable training outcomes across diverse cattle production systems, particularly in tropical and subtropical regions where *Bos indicus* genetics predominate.

### 5.5. Accessibility, Standardization, and Collaborative Development

Democratizing access to quality simulation training requires parallel efforts across multiple fronts. Low-cost simulator models such as SIMCA-COW demonstrate that effective training tools need not entail prohibitive investment [27], though balancing cost reduction with adequate fidelity remains a persistent design challenge. Open-source development initiatives—including systematic sharing of construction plans, validation data, and competency-driven assessment rubrics—could facilitate adoption in resource-constrained institutions, particularly in low- and middle-income countries.

Robust collaboration between industry, academic institutions, and professional veterinary organizations is fundamental to advancing these innovations while ensuring that technological development aligns with pedagogical principles, animal welfare standards, and practitioner needs. In the near term, systematic validation of existing simulators through rigorous educational research should take precedence over development of entirely new platforms. Validation studies must extend beyond immediate performance metrics to examine long-term skill retention, clinical transfer efficacy, and development of ethical competencies—clarifying how simulation-based training contributes to humane, sustainable veterinary practice aligned with One Health and One Welfare frameworks [43].

### 5.6. Synthesis and Implementation Priorities

The future of simulation in veterinary education depends on integrating technological sophistication with pedagogical rigor, economic accessibility, and ethical responsibility. Immediate priorities include the following: (1) systematic validation of existing simulator platforms through controlled educational research; (2) establishment of consensus standards for simulator development, testing, and performance reporting; (3) expansion of anatomically diverse models representing global cattle populations; (4) collaborative development of affordable, open-source designs for resource-limited contexts; and (5) integration of emerging technologies (AI, VR/AR, advanced haptics) only when evidence demonstrates clear pedagogical benefit relative to implementation costs.

Progress requires coordinated efforts across stakeholders to establish evidence-based guidelines that inform both development and adoption of simulation technologies across diverse educational contexts, ensuring that innovation serves—rather than displaces—fundamental pedagogical objectives and ethical commitments to animal welfare.

## 6. Final Considerations and Recommendations

This scoping review demonstrates that simulators occupy an increasingly important role in bovine reproductive training within animal and veterinary education, and in the preparation of non-graduate technical professionals. When strategically integrated into curricula, simulators support the development of technical proficiency, enhance learner confidence, and contribute to animal welfare protection aligned with the 3Rs framework—Replacement (substituting live animals where possible), Reduction (decreasing animal numbers required), and Refinement (minimizing animal suffering) [44].

Simulators support the 3Rs framework in veterinary education through three mechanisms. First, they replace initial live animal training experiences, enabling students to acquire basic orientation, hand positioning, and procedural sequencing without causing harm, with meta-analytical evidence supporting comparable learning outcomes [23]. Second, simulator-trained students require fewer live-animal sessions to achieve competency, thereby reducing overall teaching herd use [16,21,27]. Third, simulators refine live-animal use by ensuring more advanced learners—rather than complete novices—progress to clinical practice, improving handling efficiency, reducing procedural time, and minimizing repeated failed attempts [27]. However, while simulation-based training aligns conceptually with the 3Rs framework by reducing early exposure of animals to novice procedures, empirical evidence demonstrating quantifiable ethical outcomes—such as measurable reductions in animal use or stress—remains limited [45]. Ethical benefits should therefore be regarded as plausible and context-dependent rather than universally established.

The evidence indicates that simulators are most effective when functioning as foundational tools within a staged learning progression: theoretical instruction → simulator-based practice → supervised live-animal experience. Simulators enable educators to design structured, low-stakes practice environments that reduce performance anxiety, permit repeated procedural rehearsal, and allow error-based learning without animal welfare consequences. When combined with individualized feedback and standardized competency assessment methods such as OSCEs, simulator training demonstrably improves students’ technical proficiency, confidence levels, and preparedness for clinical practice.

However, simulators represent preparatory tools rather than complete educational solutions. Their optimal value depends on integration into comprehensive curricular frameworks that progress systematically from simulation to clinical practice, maintain explicit focus on animal welfare ethics and professional identity formation, and establish clear competency criteria for advancement between learning stages.

### 6.1. Limitations of Current Evidence

Important limitations characterize the current simulator literature. First, most published validation efforts remain limited to face validity and user satisfaction assessments; rigorous evidence of learning transfer to clinical performance is sparse. Second, heterogeneity in study designs, simulator types, and outcome measures complicates direct comparison and synthesis of findings. Particularly, differences in intervention design—including training sequence, total training duration, order of simulator introduction, and learner background—complicate direct comparison between studies. Moreover, the absence of industry standardization and variability in manufacturing quality further hinder cross-platform comparisons. Third, and most importantly, long-term follow-up data are scarce. All but one study [33] include follow-up measures extending beyond the immediate post-training period. Thus, retention and transfer have been infrequently assessed, limiting conclusions about skill retention and early-career performance. Fourth, the current simulator landscape demonstrates geographic and anatomical bias, predominantly representing *Bos taurus* systems and institutions in high-income countries. These limitations underscore the need for expanded, rigorous validation research across diverse educational contexts and cattle production systems.

### 6.2. Recommendations for Current Implementation

For veterinary educators, the primary take-home message of this review is that simulators are most effective when embedded intentionally within a staged curriculum, rather than used as ad hoc or standalone teaching tools. For educators and institutions seeking to implement or optimize simulation-based training with available tools, we recommend:

- Establishing clear curricular placement. Position simulator sessions early in students’ procedural learning, following theoretical instruction but preceding live-animal practice. This sequencing optimizes confidence-building and skill consolidation while maintaining animal welfare protection.
- Implementing structured feedback mechanisms. Ensure that simulator sessions include systematic feedback—whether instructor-provided, peer-delivered, or technology-enabled—paired with opportunities for immediate procedural re-rehearsal. This deliberate practice approach accelerates competency development.
- Applying standardized competency assessment. Use simulators to establish objective performance benchmarks through reproducible assessment methods (e.g., OSCE formats), enabling transparent progression criteria and evidence-based advancement decisions.
- Integrating ethical and professional development. Link simulator-based technical training with explicit curricular discussions connecting procedural competence to animal welfare responsibilities, ethical decision-making, and professional reflection. This integration cultivates practitioners who are both technically proficient and ethically grounded.
- Adapting to institutional context. Select simulator types and implementation models matched to institutional resources, learner populations, and specific procedural priorities. Cost-effective models with adequate fidelity may be as educationally effective as high-cost alternatives in appropriate contexts.

### 6.3. Research Priorities

To address evidence gaps and advance simulation-based education in bovine reproduction, we identify the following research imperatives:

- Rigorous validation studies examining learning transfer and long-term skill retention across diverse simulator platforms, learner populations, and institutional contexts;
- Comparative effectiveness research, measuring simulator training outcomes against alternative instructional approaches and identifying which learner populations or procedural competencies benefit most from simulation;
- Implementation of studies investigating how simulator integration affects curriculum quality, learning outcomes, and animal resource utilization across different educational settings;
- Ethical competency assessment, examining whether simulation-based training contributes to development of ethical reasoning and animal welfare consciousness alongside technical skill acquisition;
- Global evidence synthesis, expanding published validation data from underrepresented regions and cattle production systems to inform equitable simulator development and adoption.

### 6.4. Conclusions

Simulators represent valuable educational instruments for bovine reproductive training when deployed thoughtfully within evidence-based curricular frameworks. The available evidence supports their use as foundational tools that reduce animal welfare burden in teaching contexts, enhance student learning outcomes, and prepare practitioners for competent clinical practice. Simulators are neither solutions nor peripheral innovations—they occupy a strategic intermediate position in contemporary animal and veterinary education, complementing but not replacing live-animal experience.

Realizing the full educational potential of simulation requires sustained commitment to evidence-based implementation, rigorous validation of educational claims, and ethical integration of technical skill development with broader professional formation. Collaborative efforts among educators, researchers, and institutions are essential to advancing simulation-based training quality, expanding global access, and ensuring these tools serve the dual imperatives of clinical excellence and animal welfare protection.

## Figures and Tables

**Table 1:** Inclusion and exclusion criteria used in the study.

Inclusion Criteria	Exclusion Criteria
Original research articles, theses or dissertations	Review articles
Studies describing simulator development, implementation, validation, or evaluation in educational settings	Studies focused on basic reproduction research without educational component
Publications on teaching bovine reproductive skills using simulators	Publications outside reproduction domain or unrelated to targeted species
Studies comparing different simulators for training bovine reproductive procedures	Conference abstracts, posters, book chapters without complete original data
Peer-reviewed publications (preferred)	Studies limited to live-animal or cadaver-based teaching without simulators
Full-text availability	
No language restrictions (studies in languages other than English, Spanish, or Portuguese were translated for screening when necessary	

**Table 2:** Overview of the main simulators used for bovine reproductive training, their typological classification, design characteristics, and educational applications reported in the literature.

Simulator/Model	Type and Fidelity	Main Components/Features	Approx. Cost	Educational Application(s)	References
**Breed’n Betsy** **(Brad Pickford, Byaduk, Victoria,** **Australia)**	Physical/Mechanical Low–Medium fidelity	Steel or aluminum frame with an artificial vulva and anal sphincter containing a replica of a cow’s pelvis and a suspended latex genital apparatus Allows palpation at different gestational stages by changing the latex replica of the uterus.	Not reported	Training in reproductive anatomy, transrectal palpation, and pregnancy diagnosis.	[16,21,26,33,34]
**Simulador** **Bovino F1** **(Embriovinos SAS)**	Physical Low–Medium fidelity	Life-size bovine shell (fiberglass with latex coverings) that reproduces external landmarks of a cow; latex model of the reproductive tract (uterus/ovaries/cervix).	Not reported	Training in reproductive anatomy and transrectal palpation The simulator also allows training other medical non-reproductive procedures	[35]
**SIMCA-COW** **(Portugal)**	Physical (hand-made) Moderate–High fidelity	Cow pelvis and rigid support with interchangeable slaughterhouse uteri; adaptable for palpation, AI, and ultrasonography training.	<USD 40	Low-cost model for skills lab and technician training. Training in transrectal palpation, AI and reproductive ultrasound	[27]
**Haptic Cow/PHANToM System** **(University of Glasgow, UK)**	Haptic/Hybrid (VR) High fidelity	Virtual 3D organ models with force-feedback device inside a black and white fiberglass cow. The 3D computer-generated virtual environment simulates the bovine reproductive tract, with a wide range of fertility conditions, pregnancies and some examples of pathology.	High (institutional level)	Training in reproductive anatomy, transrectal palpation, and pregnancy diagnosis.	[4,14,26]
**PHANToM** **(stand-alone)** **(University of Glasgow, UK)**	Virtual/Haptic High fidelity	PHANToM haptic arm with virtual reproductive tract interface; automated version for independent use	Not reported	Early psychomotor training and tactile orientation.	[15]
**TrAI4Nel** **(Brazil)**	Hybrid (Physical + Electronic) High fidelity	Silicone cervix (double-layer), LED feedback, reed-switch sensors, Arduino-controlled peristaltic motion Anatomically specific for *Bos indicus*.	Not reported	AI and cervical navigation training with real-time feedback.	[22,36]

AI—Artificial Inteligence; VR—Virtual reality.

**Table 3:** Educational use of simulators for bovine reproductive procedures.

Simulator(s)	Procedures Trained	Role in Curriculum	Study Population	Training Duration	Reference
**Haptic Cow/PHANToM**	Rectal palpation, reproductive tract identification	Supplement	97 inexperienced veterinary students	Not mentioned	[14]
**Haptic Cow (virtual reality, haptic feedback)**			31 veterinary students	Not mentioned	[15]
**Haptic Cow (virtual reality, automated version)**	Rectal palpation, uterus identification		16 veterinary students	Not specified	[4]
**Breed’n Betsy**	Rectal palpation, cervix/uterus/ovaries identification, pregnancy diagnosis	Substitute	17 students in Exp 1 (8 live cow, 9 simulator); 10 students in Exp 2	25 palpations in experience 1; 200 palpations through one year	[34]
**Breed’n Betsy, hybrid models**	Pregnancy diagnosis, rectal palpation, anatomical identification	Supplement	138 veterinary students, University of Pretoria	Single session	[21]
**SIMCA-COW (low-cost)**	Anatomical identification, palpation, ultrasonography, artificial insemination		8 inexperienced veterinary students	Multiple repetitions; 4 attempts for AI	[27]
**Haptic cow vs. Breed’n Betsy comparison**	Rectal palpation, cervix and uterus location		3 groups of 25 veterinary students each	Max. 60 min (with 9 min/student in the simulator. Control group only theoretical	[26]
**Simulador Bovino F1**	Identification of cervix, uterus and ovaries		42 third-year Zootechnics students (20 experimental, 22 control)	4 h plus 2 h anatomy	[35]
**TrAI4Nel**	AI training		61 trainees divided in three groups: Control (abattoir specimens plus live cows), experimental groups (abattoir specimens and simulator in a crossed-presentation order, plus living cows)	2 h of theoretical class and a total of 20 h (experimental groups) to 24 h (control groups) of practical training	[22]

Abbreviations: AI—Artificial Insemination.

**Table 4:** Educational strategies used in simulation-based interventions for bovine reproductive procedures.

Simulator(s)	Training Design/ Structure	Group Size	Instructor Involvement	Sequence with Living Animals	Feedback and Assessment Approach	Comparison Method	Reference
**Haptic Cow/PHANToM**	Sequential sessions: anatomy review → virtual palpation → supervised debrief	Not mentioned	Instructor involved	Before live sessions	Real-time haptic feedback; instructor-guided reflection	Traditional training	[14]
**Haptic Cow (virtual reality, haptic feedback)**	Structured protocol; scaffolded virtual palpation prior to live animals.	Not mentioned	Instructor involved	Before live sessions	Expert evaluation; Objective live animal skill transfer (ultrasound verified); Student surveys.	Traditional training	[15]
**Haptic Cow (virtual reality, automated version)**	Structured peer-assisted learning with trained tutors; one-on-one simulator practice.	8 per group	Independent use	Before live sessions	Pre/post questionnaires and focus groups for feedback; Staff observation of tutor performance for quality control.	No additional training control	[4]
**Breed’n Betsy**	Scaffolded practice before live-animal palpation	Not specified	Instructor inferred in experiment 1; veterinarian educator in experiment 2	Alternative to live training sessions	Instructor demonstration + peer practice	Traditional training	[34]
**Breed’n Betsy, hybrid models**	Structured labs within reproductive module	Cohorts	Facilitator coordination	Before or with live sessions	Stepwise tasks; instructor scoring with standardized rubric	Living cow training	[21]
**SIMCA-COW (low-cost)**	Short practical course; repetition until proficiency	8 students	Initial guidance, then independent	Before live sessions	Peer feedback; timed trials (observational)	Traditional training	[27]
**Haptic cow vs. Breed’n Betsy comparison**	Short intensive workshop (2 days)	75 students in total; training cohorts of 3–5	Instructor involved	Before live sessions	Immediate instructor feedback; self-assessment forms	Theoretical instruction only; different simulators	[26]
**Simulador Bovino F1**	Scaffolded practice before live-animal palpation	Maximum 5 students per group	Instructor involved	Before live sessions	Instructor feedback in simulator groups; Expert live animal assessment (anatomical accuracy).	Traditional training	[35]
**TrAI4Nel**	Scaffolded practice before live-animal IA	multiple cohorts of 10 trainees	Instructor involved	Before live sessions	Automated LED/sensor feedback; instructor feedback	Traditional training (abattoir specimens before live cows)	[22]

Abbreviations: AI—Artificial Insemination.

**Table 5:** Summary of formal validation of available simulators to train bovine reproductive procedures.

Simulator/Model	Reported Validation Evidence	Selected References
**Breed’n Betsy**	*Face and Construct Validity:* Differentiated novice vs. experienced users; limited realism for early pregnancies.	[21,25,33,34]
**Simulador Bovino F1**	*Without Reported Validation*	[35]
**SIMCA-COW**	*Face, Content and Concurrent Validity:* Anatomical realism confirmed by experts; reduced time to complete AI; increased self-confidence.	[27]
**Haptic Cow/PHANToM System**	*Face, Content and Criterion Validity:* Improved uterine-structure identification; increased confidence and satisfaction.	[14,15]
**PHANToM** **(stand-alone)**	*Face Validity:* 97% of users reported increased confidence; supports skill transfer.	[4]
**TrAI4Nel**	*Face, Content, Construct and Criterion Validity:* Improved success rate with live animals (78.6% vs. 52.6% controls).	[22]

## Data Availability

Data sharing is not applicable to this article, as no new data was generated.

## Supplementary Materials

The following supporting information can be downloaded at: https://www.mdpi.com/article/10.3390/ani16010140/s1. Table S1: List of extracted articles for this scoping review.
