# Supplementary material for: The Use of Simulators in Training for Bovine Reproductive Procedures: A Scoping Review
Source: Animals (Basel). 2026 Jan 4;16(1):140. doi: 10.3390/ani16010140 (PMC12785041; doi:10.3390/ani16010140)
Supplement: Supplementary file 1 [file animals-16-00140-s001.zip › animals-4044799-supplementary.pdf]

Supplementary table 1. List of extracted articles for this scoping review

Reference	Reference number
Baillie, S.; Crossan, A.; Brewster, S.A.; May, S.A.; Mellor, D.J. Evaluating an automated haptic simulator designed for veterinary students to learn bovine rectal palpation. Simul Healthc 2010, 5, 261–266, doi:10.1097/SIH.0b013e3181e369bf.	4
Baillie, S.; Shore, H.; Gill, D.; May, S.A. Introducing peer-assisted learning into a veterinary curriculum: a trial with a simulator. J Vet Med Educ 2009, 36, 174–179, doi:10.3138/jvme.36.2.174.	13
Baillie, S.; Mellor, D.J.; Brewster, S.A.; Reid, S.W. Integrating a bovine rectal palpation simulator into an undergraduate veterinary curriculum. J Vet Med Educ 2005, 32, 79–85.	14
Baillie, S. Validation of the Haptic Cow: A simulator for training veterinary students. University of Glasgow, Glasgow, 2007.	15
Annandale, A.; May, C.E.; van der Leek, M.L.; Fosgate, G.T.; Kremer, W.D.; Bok, H.G.; Holm, D.E. Effect of a high-intensity one-week training programme and student-level variables on the bovine transrectal palpation and pregnancy diagnosis skills of final-year veterinary students. Vet Rec 2020, 187, e99, doi:10.1136/vr.105909.	16
Annandale, A.; Annandale, C.H.; Fosgate, G.T.; Holm, D.E. Training Method and Other Factors Affecting Student Accuracy in Bovine Pregnancy Diagnosis. J Vet Med Educ 2018, 45, 224–231, doi:https://doi.org/10.3138/jvme.1016-166r1.	21
Azuaga-Filho, H.; Santos, A.; Colaço, B.; Payan-Carreira, R. Comprehensive Validation of the TrAI4Nel Simulator for Nelore Artificial Insemination Training: A Controlled Study. Animals (Basel) 2025, 15, doi:10.3390/ani15202982	22
Annandale, A. Thinking differently about clinical skills training: The bovine pregnancy diagnosis via transrectal palpation showcase. Utrecht University, Utrecht, 2020	25
Giese, H.; Ehlers, J.P.; Gundelach, Y.; Geuenich, K.; Dilly, M. [Effects of different training methods for palpation per rectum of internal genital organs on learning success and self-evaluation of students]. Berl Munch Tierarztl Wochenschr 2016, 129, 216–224.	26
da Silva, C.B.; Pinto, E.M. Efficacy validation of a low-cost handmade simulator (SIMCA-COW) in palpation, ultrasonography evaluation, and artificial insemination in cows. Vet World 2023, 16, 144–148, doi:https://doi.org/10.14202/vetworld.2023.144-148	27
Annandale, A.; Fosgate, G.T.; Bok, H.; Holm, D.E. Ability of a bovine transrectal palpation objective structured clinical examination to predict veterinary students' pregnancy diagnosis accuracy. Vet Rec 2019, 185, 171, doi:10.1136/vr.105022	33
Bossaert, P.; Leterme, L.; Caluwaerts, T.; Cools, S.; Hostens, M.; Kolkman, I.; de Kruif, A. Teaching transrectal palpation of the internal genital organs in cattle. J Vet Med Educ 2009, 36, 451–460, doi:https://doi.org/10.3138/jvme.36.4.451	34
Baracaldo-Martinez, A.; Domínguez-Castaño, P.; Franco-Hernández, E.N.; Atuesta-Bustos, J.E.; Robayo-Triviño, D.A. Uso de un simulador bovino para prácticas de palpación transrectal. Revista de Investigaciones Veterinarias del Perú 2019, 30, 1342–1346	35
